# Lactate Dehydrogenase-Derived Indices and Prognosis in Patients with Resectable Gastric Cancer

**DOI:** 10.31662/jmaj.2025-0407

**Published:** 2025-12-05

**Authors:** Masayuki Urabe, Mami Suzuki, Takahiro Fukai, Yui Hasegawa, Emi Terai, Yoshitaka Kiya, Goki Morizono, Masaya Hiyoshi, Toshiyuki Watanabe, Yojiro Hashiguchi

**Affiliations:** 1 Gastrointestinal Surgery Division, Department of Surgery, Japanese Red Cross Omori Hospital, Tokyo, Japan

**Keywords:** gastric cancer, lactate dehydrogenase, lactate dehydrogenase-to-albumin ratio, lactate dehydrogenase-to-lymphocyte ratio, survival

## Abstract

**Introduction:**

Robust methodologies for risk stratification remain necessary for gastric cancer (GC). We investigated the prognostic significance of preoperative lactate dehydrogenase (LDH) and two LDH-based indices, the LDH-to-lymphocyte ratio (LLR) and LDH-to-albumin ratio (LAR), in patients undergoing curative resection for GC.

**Methods:**

We retrospectively reviewed the medical records of 225 consecutive patients with GC who underwent R0 surgical resection. The prognostic value of preoperative LDH, LLR, and LAR was assessed using time-dependent receiver operating characteristic curves and Cox proportional hazards regression. Optimal cut-off values were determined with X-tile software.

**Results:**

The median follow-up period was 61 months. The areas under the curves for predicting overall survival (OS) and relapse-free survival (RFS) were notably higher for LLR and LAR as compared to LDH alone. In univariate Cox analyses, both LLR and LAR were significantly associated with OS and RFS, whereas LDH was not. In multivariate analyses, LLR and LAR remained independent predictors of OS (high LLR: hazard ratio [HR] 2.66, 95% confidence interval [CI] 1.34-5.28, p = 0.005; high LAR: HR 2.61, 95% CI 1.45-4.71, p = 0.001). Similarly, both indices retained independent prognostic significance for RFS (high LLR: HR 2.22, 95% CI 1.14-4.31, p = 0.019; high LAR: HR 2.65, 95% CI 1.49-4.72, p < 0.001).

**Conclusions:**

Preoperative LLR and LAR are independent prognostic indicators for OS and RFS in patients with resectable GC. These indices may facilitate early identification of high-risk patients and support individualized treatment strategies.

## Introduction

Gastric cancer (GC) remains one of the most lethal malignancies, currently ranking fifth among causes of cancer-related mortality worldwide ^(1)^. Despite advances in therapeutic strategies, improvements in prognosis have been modest, and GC continues to pose a major public health challenge ^(2)^. In the current era of multimodal treatment strategies anchored in surgical resection, pretreatment risk stratification is essential, and there is a pressing need for reliable, readily accessible tools to inform therapeutic decision-making. However, effective modalities to optimize treatment selection remain limited.

Lactate dehydrogenase (LDH) is a ubiquitous cytoplasmic enzyme that catalyzes the conversion of pyruvate to lactate during anaerobic glycolysis and is widely used in clinical practice as a marker of tissue injury. Owing to its integral role in cancer metabolism, LDH has been classically proposed as a potential prognostic biomarker in various solid tumors and hematologic malignancies ^(3), (4)^. Nevertheless, its prognostic significance in GC remains controversial ^(5), (6)^. Moreover, LDH-derived indices, such as the LDH-to-lymphocyte ratio (LLR) and LDH-to-albumin ratio (LAR) ^(7), (8), (9), (10), (11)^, have not been comprehensively evaluated in the context of GC.

We hypothesized that LDH and LDH-related indices may serve as valuable biomarkers for stratifying survival outcomes in GC. To test this hypothesis, we performed a retrospective analysis to assess their prognostic significance in patients who underwent curative resection for GC.

## Materials and Methods

### Study population

From a prospectively maintained institutional database, we identified 293 consecutive patients who underwent oncological gastrectomy with lymphadenectomy for GC between August 2010 and December 2024. Sixty-eight patients were excluded for the following reasons: R1 or R2 resection margins (n = 27), cancer of the remnant stomach (n = 9), synchronous malignancies (n = 16), receipt of neoadjuvant chemotherapy (n = 2), emergency surgery (n = 1), follow-up of less than 1 month (n = 1), or incomplete perioperative data (n = 12). No cases with cholangitis, acute infectious diseases, or connective tissue diseases were included. The medical records of the remaining 225 patients were retrospectively reviewed. This study was approved by the Institutional Review Board (number 24-33).

### Clinicopathological data

Tumor stage was assigned according to the eighth edition of the Union for International Cancer Control Tumor-Node-Metastasis classification ^(12)^. Histological subtype was categorized as intestinal or diffuse, based on Lauren’s criteria ^(13)^. Postoperative complications were defined as events occurring within 30 days of surgery and graded as Clavien-Dindo grade ≥III ^(14)^.

Postoperative surveillance was conducted in accordance with the Japanese Gastric Cancer Association guidelines, with follow-up scheduled for at least 5 years or until death ^(15)^. For patients diagnosed with pathological stage II/III disease (excluding T3N0), the introduction of adjuvant chemotherapy based on S-1 (an oral fluoropyrimidine derivative) was considered. Postoperative follow-up routinely included physical examination, esophagogastroduodenoscopy, computed tomography, abdominal ultrasonography, and blood tests. When recurrence was detected, systemic chemotherapy was initiated when clinically appropriate, based on the patient’s general condition, organ function, and extent of disease. For patients who missed scheduled visits, survival and recurrence status were confirmed by telephone interview. Surveillance for all patients was completed in July 2025. During the study period, 71 deaths occurred, comprising 28 cancer-specific and 43 non-GC-related deaths. As of the last follow-up, the median follow-up duration among survivors was 61 months.

### LDH and LDH-based indices

Preoperative blood test data were basically obtained within 7 days before surgery. The LLR was calculated as serum LDH (U/L) divided by total lymphocyte count (×10^3^/μL), and the LAR as serum LDH (U/L) divided by serum albumin (g/dL).

In addition to LDH and LDH-derived indices, the preoperative neutrophil-to-lymphocyte ratio (NLR) was evaluated as a representative prognostic marker in GC ^(16)^. NLR was calculated as the neutrophil count divided by the total lymphocyte count.

### Statistical analysis

Statistical analyses were performed using JMP Student Edition version 18.2.1 (SAS Institute, Cary, NC, USA), X-tile version 3.6.1 (Yale University, New Haven, CT, USA), and R version 4.5.1 (R Foundation for Statistical Computing, Vienna, Austria). All tests were two-tailed, and a *P* value <0.05 was considered statistically significant.

Continuous variables were compared using the Wilcoxon rank-sum test. Overall survival (OS) was defined as the interval from surgery to death from any cause, and relapse-free survival (RFS) as the interval from surgery to recurrence or death from any cause. Optimal cut-off values for LDH, LLR, LAR, and NLR were determined using X-tile plots based on survival outcomes ^(17)^. Survival curves were generated with the Kaplan-Meier method and compared using the log-rank test. Factors associated with OS and RFS were first evaluated by univariate Cox proportional hazards regression, followed by multivariate models adjusting for known prognostic variables: age at surgery, sex, type of gastrectomy, tumor depth, nodal metastasis, preoperative endoscopic resection, adjuvant chemotherapy, and preoperative NLR ^(16), (18)^. The number of variables included in the model was restricted according to the number of events to ensure stability and prevent overfitting. To mitigate potential collinearity, each index was analyzed using separate models. Time-dependent receiver operating characteristic (ROC) curves for survival prediction based on preoperative LDH, LLR, LAR, and NLR were generated using the “timeROC” function in R.

## Results

### Associations of preoperative LDH, LLR, and LAR with clinicopathological characteristics

The associations between preoperative indices and baseline clinicopathological characteristics are presented in Table 1. Preoperative LDH was significantly associated with age, dichotomized at 70 years. The LLR was significantly associated with age, tumor depth, and the occurrence of postoperative complications. The LAR demonstrated significant associations with age, gender, surgical approach, tumor depth, lymphovascular invasion, and prior endoscopic resection.

**Table 1.** Associations between Preoperative LDH-Based Indices and Clinicopathological Factors.

Characteristics	#	LDH, U/L Median (IQR)	p Value	LLR Median (IQR)	p Value	LAR Median (IQR)	p Value
Total	225	178 (155-211)		114.2 (86.1-155.9)		47.9 (40.1-57.1)	
*Age at surgery (years)*							
≤69	71	169 (148-196)	0.042^*^	99.7 (72.5-123.4)	<0.001^*^	42.8 (36.5-52.1)	<0.001^*^
≥70	154	181 (157-213)		120.0 (93.1-170.5)		50.3 (42.3-58.0)	
*Gender*							
Male	154	173 (154-200)	0.10	114.5 (82.1-156.0)	0.61	45.9 (39.5-55.5)	0.023^*^
Female	71	194 (157-213)		113.9 (92.6-156.1)		51.9 (44.7-57.6)	
*Hepatitis*							
Absent	214	177 (154-210)	0.017^*^	113.9 (86.2-153.3)	0.34	47.9 (39.8-56.6)	0.094
Present	11	197 (187-259)		149.1 (85.4-190.8)		55.4 (44.9-67.9)	
*Main histology*							
Intestinal	149	177 (156-210)	0.79	113.9 (87.3-156.4)	0.84	47.9 (39.9-57.1)	0.78
Diffuse	76	179 (153-213)		114.2 (83.1-155.1)		47.6 (40.3-57.1)	
*Type of gastrectomy*							
Partial	168	181 (159-212)	0.096	113.5 (88.4-152.9)	0.83	47.0 (40.3-56.6)	0.43
Total	57	171 (147-203)		115.8 (83.6-167.0)		50.0 (39.9-58.6)	
*Surgical approach*							
Open	186	180 (156-212)	0.19	116.0 (88.8-157.1)	0.11	49.4 (41.2-58.0)	<0.001^*^
Laparoscopic/robotic	39	169 (155-200)		107.0 (76.2-137.7)		40.9 (26.0-45.6)	
*pT classification*							
T1	96	176 (154-208)	0.46	108.5 (79.2-143.1)	0.015^*^	44.8 (38.5-53.3)	0.001^*^
T2-4	129	180 (156-213)		119.3 (92.0-167.5)		50.7 (42.0-58.7)	
*pN classification*							
N0	148	178 (156-212)	0.47	113.0 (85.5-150.1)	0.32	46.7 (39.6-57.2)	0.27
N1-3	77	179 (152-205)		116.7 (88.3-169.2)		49.4 (42.5-56.9)	
*Lymphovascular involvement*							
Absent	99	175 (156-210)	0.68	113.9 (88.3-152.4)	0.69	44.9 (39.3-54.3)	0.005^*^
Present	126	180 (155-211)		115.0 (86.0-162.5)		50.8 (42.0-58.4)	
*Preoperative ER*							
Absent	200	180 (156-212)	0.14	115.1 (89.2-156.0)	0.095	48.8 (41.2-57.6)	0.004^*^
Present	25	166 (150-191)		86.2 (68.1-167.9)		39.8 (36.7-47.9)	
*Adjuvant chemotherapy*							
Absent	189	177 (154-210)	0.55	114.3 (88.2-158.4)	0.35	47.3 (40.1-55.9)	0.29
Present	36	181 (161-216)		107.2 (82.1-141.6)		50.7 (40.0-60.9)	
*Postoperative complications*							
Absent	201	178 (156-212)	0.68	111.5 (82.4-149.8)	<0.001^*^	47.9 (39.8-56.9)	0.23
Present	24	178 (153-197)		150.8 (114.6-209.1)		47.8 (43.1-67.4)	

ER: endoscopic resection; IQR: interquartile range; LAR: lactate dehydrogenase-to-albumin ratio; LDH: lactate dehydrogenase; LLR: lactate dehydrogenase-to-lymphocyte ratio. ^*^P < 0.05.

Linear regression analyses were conducted to evaluate the associations of preoperative LDH, LLR, and LAR with NLR. No significant correlation was observed between LDH and NLR (p = 0.41, R^2^ = 0.0030). Although both LLR and LAR exhibited significant associations with NLR (LLR: p < 0.001, R^2^ = 0.31; LAR: p = 0.021, R^2^ = 0.024), the strength of these correlations was weak, as indicated by low R^2^ values.

### Time-dependent ROC analyses

**Figure 1.**
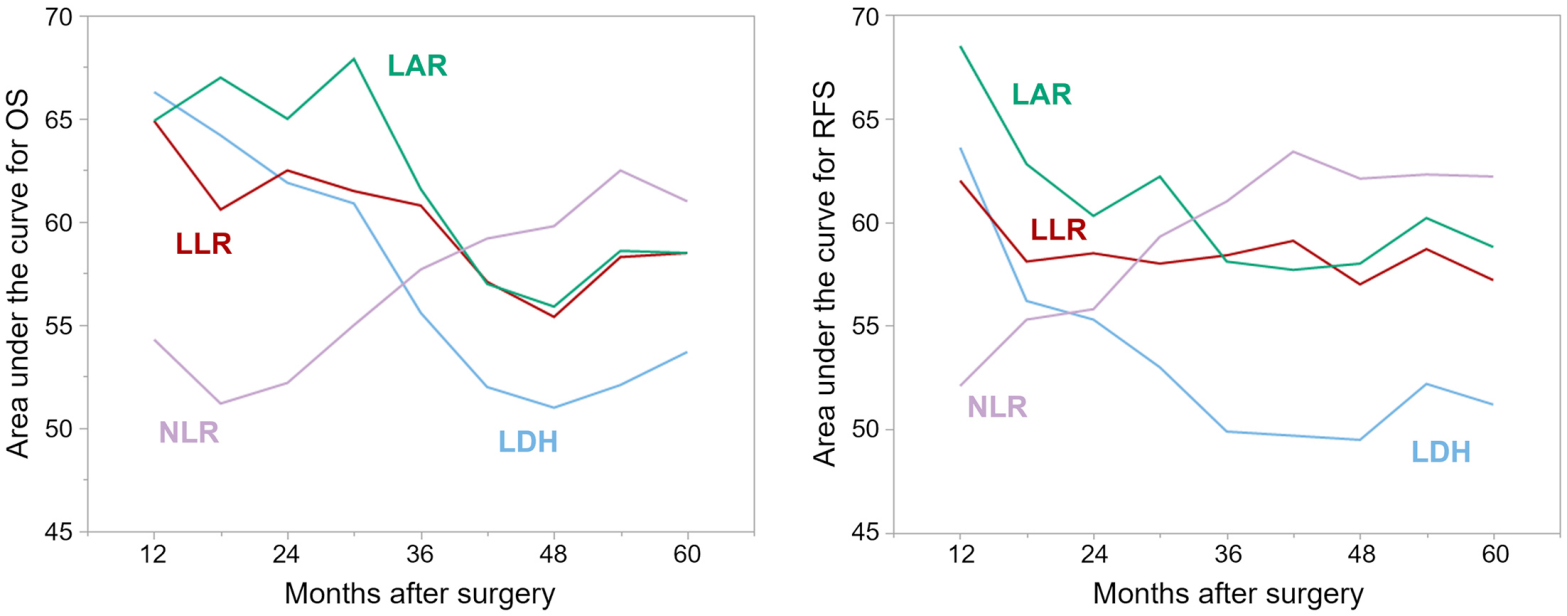
Time-dependent ROC analyses for overall and relapse-free survival estimations. ROC: receiver operating characteristic.

Time-dependent ROC analyses were performed, and the area under the curve values for each index were plotted over time to compare their prognostic performance (Figure 1). The preoperative LLR and LAR demonstrated superior predictive accuracy for both OS and RFS compared to LDH. Furthermore, the prognostic performance of LLR and LAR was largely comparable to that of the preoperative NLR. The prognostic accuracy of preoperative LLR and LAR was particularly high during the relatively early postoperative period, within 3 years after surgery.

### Determination of cut-off points and survival curve analyses

**Figure 2.**
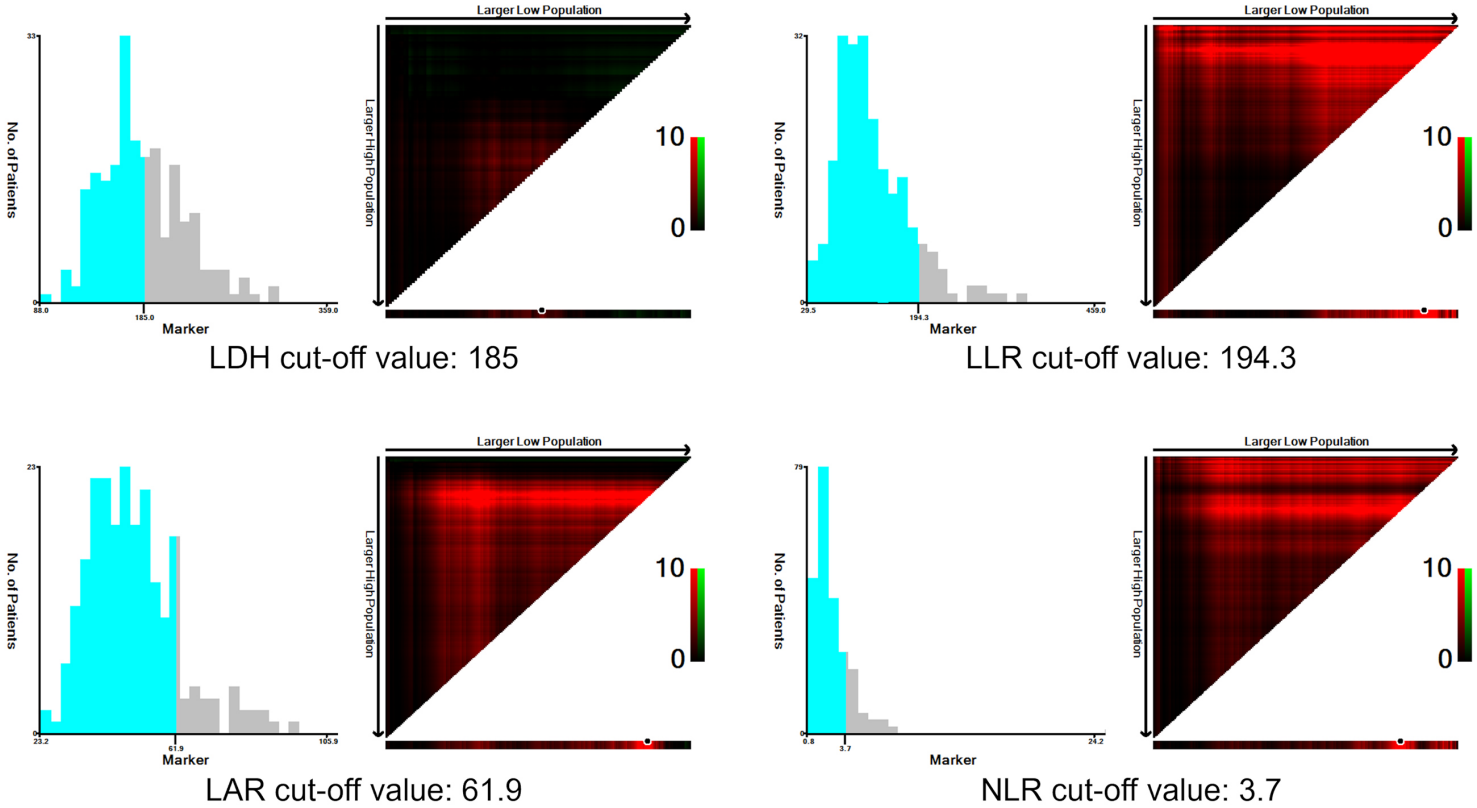
Determination of cut-off values using X-tile software.

Optimal cut-off values for each index were determined using X-tile software, based on 5-year survival data from the entire patient cohort. The resulting cut-off values were 185 U/L for LDH, 194.3 for LLR, 61.9 for LAR, and 3.7 for NLR (Figure 2).

**Figure 3.**
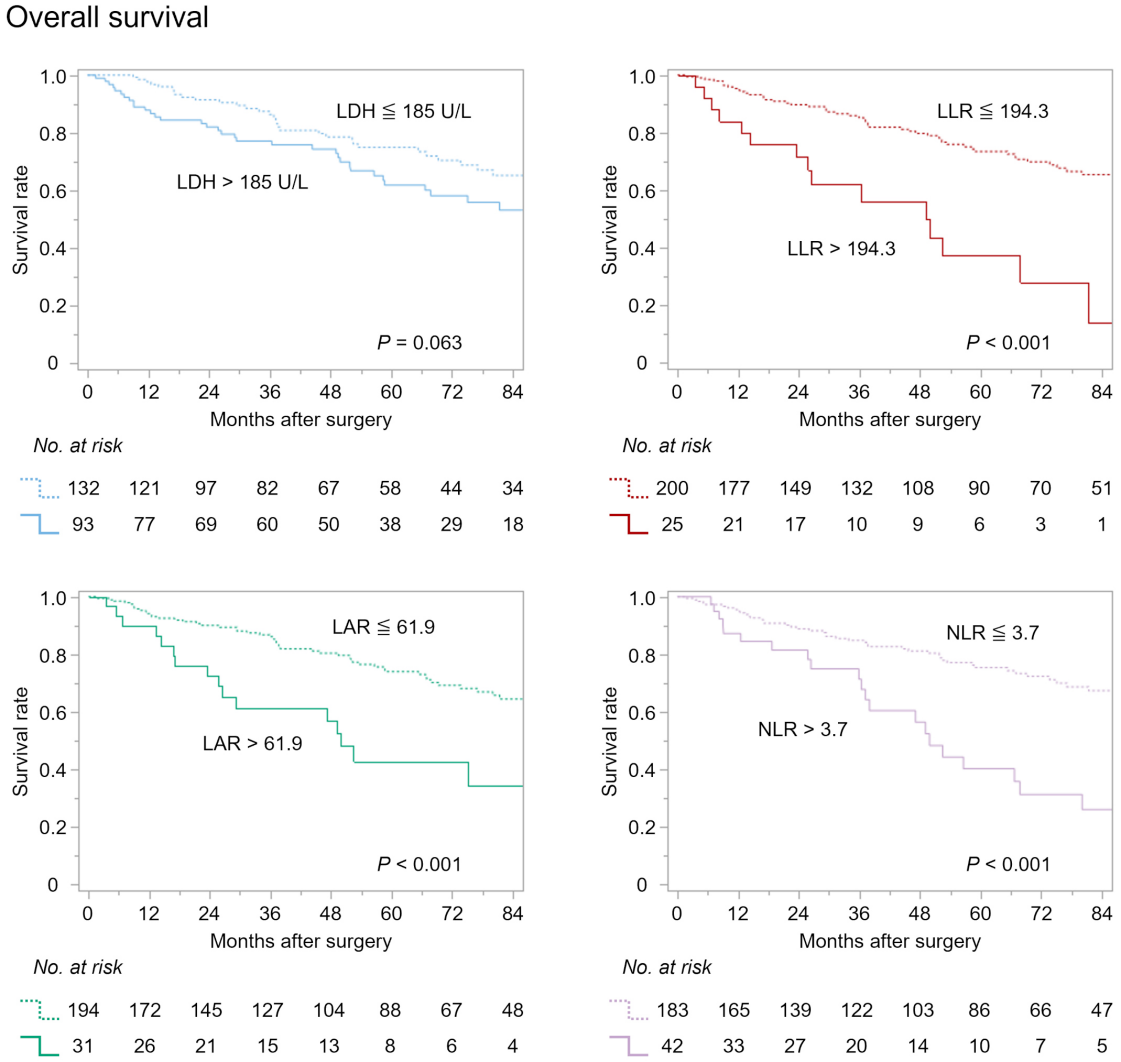
Overall survival curves according to preoperative LDH, LLR, LAR, and NLR. LAR: LDH-to-albumin ratio; LDH: lactate dehydrogenase; LLR: LDH-to-lymphocyte ratio; NLR: neutrophil-to-lymphocyte ratio.

**Figure 4.**
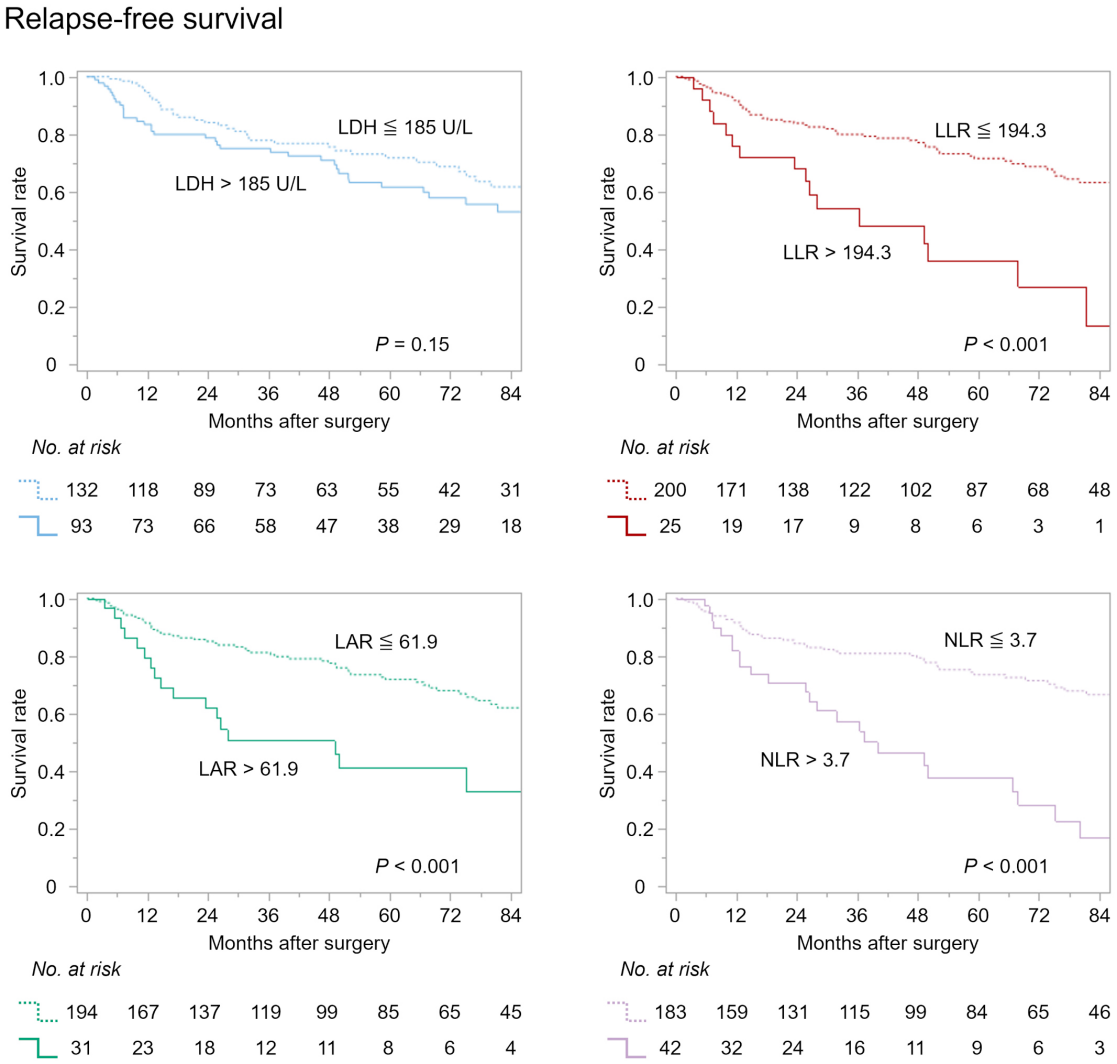
Relapse-free survival curves according to preoperative LDH, LLR, LAR, and NLR. LAR: LDH-to-albumin ratio; LDH: lactate dehydrogenase; LLR: LDH-to-lymphocyte ratio; NLR: neutrophil-to-lymphocyte ratio.

Patients were stratified into high and low groups according to these thresholds, and Kaplan-Meier survival analyses were conducted for both OS (Figure 3) and RFS (Figure 4). While the preoperative LDH level did not significantly stratify OS (p = 0.063), LLR, LAR, and NLR all demonstrated significant prognostic discrimination for OS (all p < 0.001). Similarly, RFS was significantly stratified by preoperative LLR, LAR, and NLR (all p < 0.001), whereas LDH failed to show a statistically significant association (p = 0.15).

### Univariate and multivariate Cox regression analyses

Univariate Cox regression analyses showed that preoperative LLR, LAR, and NLR were significantly associated with both OS and RFS (all p < 0.001), whereas preoperative LDH was not (Table 2). In multivariate analysis, both LLR and LAR emerged as independent predictors of OS (high LLR: hazard ratio [HR] 2.66, 95% confidence interval [CI] 1.34-5.28, p = 0.005) (high LAR: HR 2.61, 95% CI 1.45-4.71, p = 0.001), while LDH remained non-significant (Table 3). For RFS, LLR, and LAR also retained independent prognostic value in the multivariate model (high LLR: HR 2.22, 95% CI 1.14-4.31, p = 0.019) (high LAR: HR 2.65, 95% CI 1.49-4.72, p < 0.001), whereas LDH again was not significant (Table 4).

**Table 2.** Univariate Cox Regression Analyses for Overall and Relapse-Free Survival Rates.

Characteristics	Overall survival		Relapse-free survival	
	HR (95% CI)	p Value	HR (95% CI)	p Value
Age at surgery (≥70 years)	2.39 (1.32-4.31)	0.004^*^	1.95 (1.13-3.36)	0.016^*^
Gender (male)	1.89 (1.07-3.33)	0.029^*^	1.95 (1.12-3.38)	0.018^*^
Hepatitis (present)	3.34 (1.53-7.31)	0.003^*^	2.84 (1.31-6.20)	0.009^*^
Main histology (intestinal)	1.35 (0.81-2.25)	0.25	1.35 (0.83-2.20)	0.23
Type of gastrectomy (total)	2.17 (1.34-3.50)	0.002^*^	2.13 (1.34-3.37	0.001^*^
Surgical approach (open)	1.90 (0.82-4.39)	0.13	1.84 (0.84-4.00)	0.13
pT classification (T2-4)	3.01 (1.72-5.26)	<0.001^*^	3.23 (1.88-5.54)	<0.001^*^
pN classification (N1-3)	2.28 (1.43-3.63)	<0.001^*^	2.56 (1.63-4.01)	<0.001^*^
Lymphovascular involvement (present)	2.30 (1.37-3.87)	0.002^*^	2.70 (1.62-4.51)	<0.001^*^
Preoperative ER (present)	0.46 (0.15-1.47)	0.19	0.38 (0.12-1.22)	0.10
Adjuvant chemotherapy (present)	1.70 (0.98-2.94)	0.057	1.90 (1.11-3.22)	0.018^*^
Postoperative complications (present)	2.18 (1.17-4.06)	0.014^*^	1.87 (1.01-3.47)	0.046^*^
Preoperative LDH (>185 U/L)	1.55 (0.97-2.47)	0.065	1.38 (0.88-2.17)	0.15
Preoperative LLR (>194.3)	3.30 (1.85-5.88)	<0.001^*^	2.97 (1.70-5.19)	<0.001^*^
Preoperative LAR (>61.9)	2.68 (1.55-4.63)	<0.001^*^	2.61 (1.53-4.43)	<0.001^*^
Preoperative NLR (>3.7)	2.92 (1.76-4.86)	<0.001^*^	3.31 (2.03-5.38)	<0.001^*^

CI: confidence interval; ER: endoscopic resection; HR: hazard ratio; LAR: lactate dehydrogenase-to-albumin ratio; LDH: lactate dehydrogenase; LLR: lactate dehydrogenase-to-lymphocyte ratio; NLR: neutrophil-to-lymphocyte ratio. ^*^p < 0.05.

**Table 3.** Multivariate Cox Regression Analyses for Overall Survival.

Model for LDH		
Characteristics	Overall survival	
	Adjusted HR (95% CI)	p Value
Age at surgery (≥70 years)	2.83 (1.51-5.30)	0.001*
Gender (male)	2.59 (1.40-4.80)	0.003*
Type of gastrectomy (total)	1.40 (0.82-2.41)	0.22
pT classification (T2-4)	2.16 (1.13-4.12)	0.020*
pN classification (N1-3)	1.54 (0.79-3.00)	0.21
Preoperative ER (present)	0.58 (0.18-1.93)	0.38
Adjuvant chemotherapy (present)	0.87 (0.43-1.76)	0.70
Preoperative NLR (>3.7)	1.84 (1.07-3.16)	0.027*
Preoperative LDH (>185 U/L)	1.53 (0.94-2.48)	0.084
		
**Model for LLR**		
**Characteristics**	**Overall survival**	
	**Adjusted HR (95% CI)**	**p Value**
Age at surgery (≥70 years)	2.82 (1.51-5.24)	0.001*
Gender (male)	2.58 (1.39-4.77)	0.003*
Type of gastrectomy (total)	1.53 (0.89-2.61)	0.12
pT classification (T2-4)	2.18 (1.13-4.21)	0.020*
pN classification (N1-3)	1.47 (0.76-2.86)	0.25
Preoperative ER (present)	0.43 (0.12-1.47)	0.18
Adjuvant chemotherapy (present)	0.94 (0.47-1.88)	0.86
Preoperative NLR (>3.7)	1.34 (0.73-2.43)	0.34
Preoperative LLR (>194.3)	2.66 (1.34-5.28)	0.005*
		
**Model for LAR**		
**Characteristics**	**Overall survival**	
	**Adjusted HR (95% CI)**	**p Value**
Age at surgery (≥70 years)	2.95 (1.58-5.48)	< 0.001*
Gender (male)	2.63 (1.42-4.89)	0.002*
Type of gastrectomy (total)	1.29 (0.75-2.22)	0.37
pT classification (T2-4)	2.13 (1.10-4.12)	0.025*
pN classification (N1-3)	2.05 (1.02-4.10)	0.044*
Preoperative ER (present)	0.61 (0.18-2.03)	0.42
Adjuvant chemotherapy (present)	0.76 (0.38-1.55)	0.45
Preoperative NLR (>3.7)	1.68 (0.98-2.88)	0.060
Preoperative LAR (>61.9)	2.61 (1.45-4.71)	0.001*

CI: confidence interval; ER: endoscopic resection; HR: hazard ratio, LAR: lactate dehydrogenase-to-albumin ratio; LDH: lactate dehydrogenase; LLR: lactate dehydrogenase-to-lymphocyte ratio; NLR: neutrophil-to-lymphocyte ratio. *p < 0.05.

**Table 4.** Multivariate Cox Regression Analyses for Relapse-Free Survival.

Model for LDH		
Characteristics	Relapse-free survival	
	Adjusted HR (95% CI)	p Value
Age at surgery (≥70 years)	2.17 (1.22-3.84)	0.008*
Gender (male)	2.58 (1.43-4.68)	0.002*
Type of gastrectomy (total)	1.26 (0.75-2.12)	0.38
pT classification (T2-4)	2.13 (1.14-3.99)	0.018*
pN classification (N1-3)	1.69 (0.90-3.18)	0.11
Preoperative ER (present)	0.52 (0.16-1.72)	0.29
Adjuvant chemotherapy (present)	0.91 (0.46-1.78)	0.78
Preoperative NLR (>3.7)	2.02 (1.20-3.40)	0.009*
Preoperative LDH (>185 U/L)	1.40 (0.88-2.23)	0.15
		
**Model for LLR**		
**Characteristics**	**Relapse-free survival**	
	**Adjusted HR (95% CI)**	**p Value**
Age at surgery (≥70 years)	2.15 (1.22-3.79)	0.008*
Gender (male)	2.55 (1.41-4.61)	0.002*
Type of gastrectomy (total)	1.36 (0.81-2.28)	0.25
pT classification (T2-4)	2.13 (1.13-4.02)	0.020*
pN classification (N1-3)	1.62 (0.86-3.02)	0.13
Preoperative ER (present)	0.41 (0.12-1.38)	0.15
Adjuvant chemotherapy (present)	0.96 (0.50-1.87)	0.92
Preoperative NLR (>3.7)	1.53 (0.86-2.74)	0.15
Preoperative LLR (>194.3)	2.22 (1.14-4.31)	0.019*
		
**Model for LAR**		
**Characteristics**	**Relapse-free survival**	
	**Adjusted HR (95% CI)**	**p Value**
Age at surgery (≥70 years)	2.10 (1.18-3.72)	0.011*
Gender (male)	2.59 (1.43-4.69)	0.002*
Type of gastrectomy (total)	1.13 (0.67-1.91)	0.65
pT classification (T2-4)	2.11 (1.11-3.99)	0.022*
pN classification (N1-3)	2.25 (1.16-4.34)	0.016*
Preoperative ER (present)	0.55 (0.17-1.83)	0.33
Adjuvant chemotherapy (present)	0.79 (0.40-1.55)	0.50
Preoperative NLR (>3.7)	1.86 (1.10-3.12)	0.020*
Preoperative LAR (>61.9)	2.65 (1.49-4.72)	<0.001*

CI: confidence interval; ER: endoscopic resection; HR: hazard ratio, LAR: lactate dehydrogenase-to-albumin ratio; LDH: lactate dehydrogenase; LLR: lactate dehydrogenase-to-lymphocyte ratio; NLR: neutrophil-to-lymphocyte ratio. *p < 0.05.

## Discussion

In this study, we retrospectively evaluated the association between preoperative LDH, LDH-derived indices, and survival outcomes in patients with resectable GC. LDH alone did not exhibit a significant association with long-term outcomes. However, two composite indices, which integrate LDH with total lymphocyte count and albumin level, respectively, demonstrated significant prognostic relevance. Notably, their predictive performance was comparable to that of the conventional NLR, and, in multivariate analysis, both LLR and LAR overperformed NLR in terms of independent prognostic power.

LAR has been widely recognized as a predictor of outcomes in cardiovascular, cerebrovascular, and respiratory diseases ^(7), (8), (9)^. In contrast, evidence linking LAR to cancer prognosis remains limited. For GC specifically, we identified only a single study, which included a highly limited sample size (n = 81) ^(19)^. Regarding LLR, it has been described as a prognostic marker in renal cell carcinoma and diffuse large B-cell lymphoma ^(10), (11)^, but no prior study has evaluated its relevance in GC. To our knowledge, this is the first study to demonstrate that preoperative LLR independently predicts prognosis in patients with resectable GC.

LDH is well recognized as an indicator of non-specific tissue injury, but it also plays a pivotal role in anaerobic metabolism, a hallmark of cancer cells driven by enhanced glycolytic activity. LDH has been reported as both a prognostic biomarker and a tool for monitoring therapeutic response in various malignancies ^(3), (4)^. Additionally, elevated LDH levels correlate with higher infiltration of immune cells, including macrophages, neutrophils, T helper cells, tumor-infiltrating lymphocytes, and regulatory T cells, suggesting a link with the immune-inflammatory microenvironment ^(3), (20)^. Despite this biological plausibility, the prognostic significance of LDH in GC remains controversial ^(5), (6)^. In our analysis, LDH alone exhibited only a modest, non-significant association with survival. By contrast, when integrated with lymphocyte count or albumin, LDH-based indices demonstrated substantially stronger prognostic performance. Intriguingly, when we assessed their association with cancer-specific survival versus non-GC-related mortality, neither LLR nor LAR showed strong correlations with cancer-specific outcomes; instead, both were significantly associated with non-GC-related deaths (Supplementary Figure S1). Our time-dependent ROC analyses demonstrated that LLR and LAR exhibited particularly high prognostic accuracy during the relatively early postoperative period (Figure 1). These observations suggest that LDH, as a surrogate for non-specific tissue damage, systemic inflammation, and immune dysregulation, in combination with nutritional status, may help identify patients at elevated risk of early-phase mortality from non-cancer-related causes.

Routine measurement of LDH and calculation of LDH-based indices could facilitate the identification of high-risk patients and enable more individualized treatment strategies. Notably, elderly patients with GC are highly susceptible to non-cancer-related mortality ^(21)^. In the current era, where the incidence of elderly GC cases is increasing at an alarming rate ^(22)^, identifying factors such as LLR and LAR that can predict such outcomes holds considerable clinical significance. Patients with elevated LLR or LAR may warrant special consideration for surgical safety rather than oncological radicality, such as minimizing the extent of lymphadenectomy or tailoring the resection range. Thus, LDH-based markers may serve as valuable tools to guide risk-adapted treatment planning.

This study has limitations. First, its retrospective, single-institution design could introduce the potential for selection bias. Second, the study period (2010-2024) encompassed substantial changes in clinical practice, including widespread adoption of minimally invasive approaches, implementation of enhanced recovery after surgery protocols, and significant advances in perioperative systemic therapy ^(2)^. Third, the competing risk of non-GC-related deaths may have led to an underestimation of disease-specific prognostic associations. Fourth, while dichotomization based on cut-off values is a common approach in clinical research, it can lead to loss of statistical power and insufficient control of confounding factors ^(23)^. These factors may have influenced outcomes. Validation in larger, prospective, multicenter cohorts is warranted.

In summary, preoperative LLR and LAR were identified as independent predictors of survival outcomes in patients undergoing radical surgery for GC. These parameters may facilitate early-phase risk stratification in resectable GC cases and contribute to the optimization of tailored treatment strategies.

## Article Information

### Author Contributions

Masayuki Urabe contributed to the conception and design of the study. All authors acquired data. Masayuki Urabe performed data interpretation and drafted the manuscript. Yoshitaka Kiya, Goki Morizono, Masaya Hiyoshi, Toshiyuki Watanabe, and Yojiro Hashiguchi critically revised the manuscript. All authors read and approved the final version prior to submission.

### Conflicts of Interest

None

### Approval by Institutional Review Board

This study was approved by the institutional ethics committee of the Japanese Red Cross Omori Hospital (Identification 24-33).

### Informed Consent

Written informed consent was waived because of the retrospective design.

## Supplement

Supplementary Material Supplementary Figure S1. Cancer-specific survival curves and non-GC-related death curves according to preoperative LDH, LLR, LAR, and NLR.
LAR: LDH-to-albumin ratio; LDH: lactate dehydrogenase; LLR: LDH-to-lymphocyte ratio; NLR: neutrophil-to-lymphocyte ratio.
