# Supplementary material for: Lactate Dehydrogenase-Derived Indices and Prognosis in Patients with Resectable Gastric Cancer
Source: JMA J. 2025 Dec 5;9(1):321–30. doi: 10.31662/jmaj.2025-0407 (PMC12889383; doi:10.31662/jmaj.2025-0407)
Supplement: Supplementary Material — Supplementary Figure S1. Cancer-specific survival curves and non-GC-related death curves according to preoperative LDH, LLR, LAR, and NLR. LAR: LDH-to-albumin ratio; LDH: lactate dehydrogenase; LLR: LDH-to-lymphocyte ratio; NLR: neutrophil-to-lymphocyte ratio. [file 2433-3298-9-1-0321-s001.pdf]

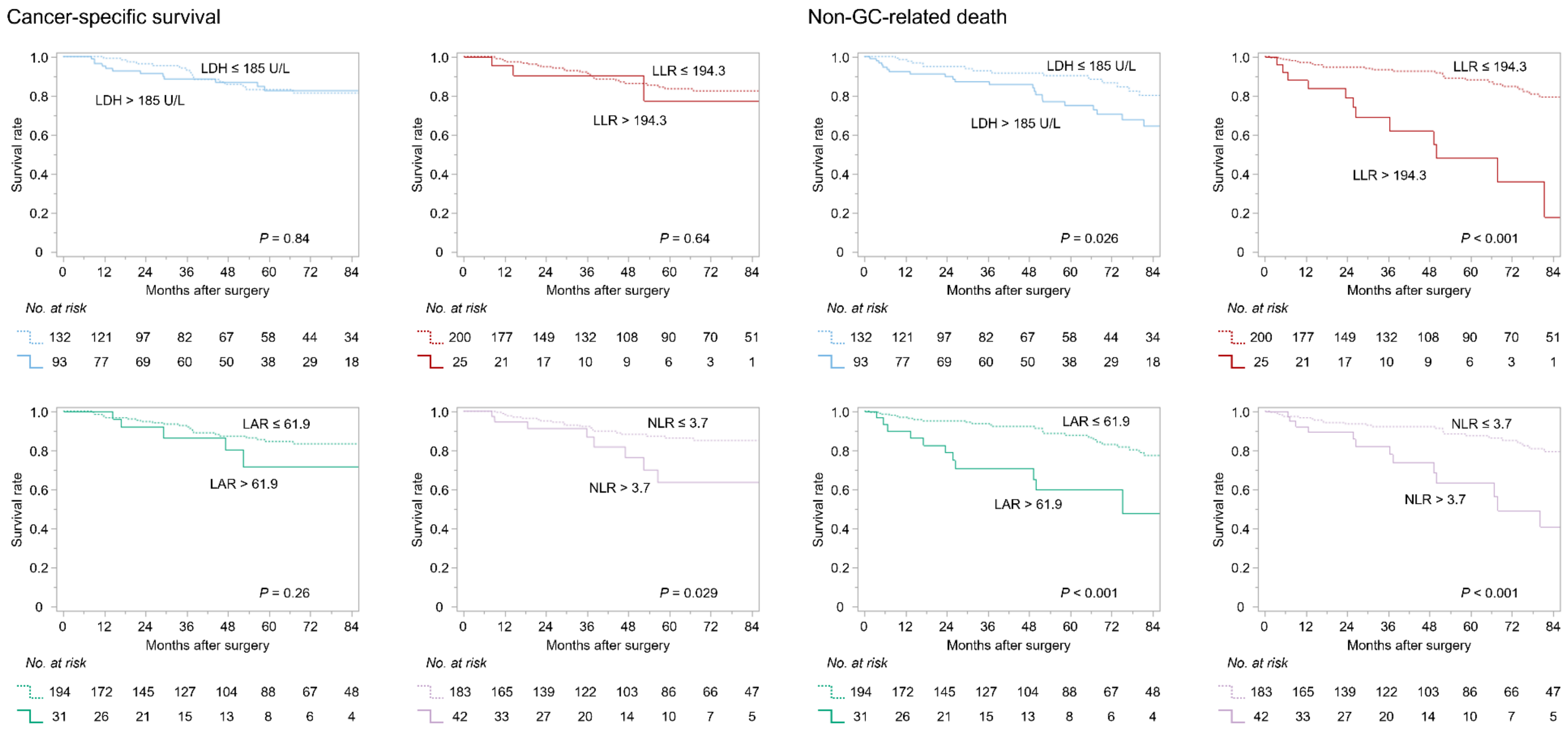

Supplementary Figure S1. Cancer-specific survival curves and non–GC-related death curves according to preoperative LDH, LLR, LAR, and NLR.
